# Fecal microbiota transplantation alleviates steatosis and inflammation in high-fat and high-sugar diet-induced fatty liver in mice

**DOI:** 10.3389/fcell.2026.1723128

**Published:** 2026-02-26

**Authors:** Fangxia Mi, Jinglu Guo, Wentao Zheng, Jianwei Shen, Hua Ye

**Affiliations:** 1 The Affiliated Lihuili Hospital of Ningbo University, Ningbo, Zhejiang, China; 2 Ningbo Hangzhou Bay Hospital (Ningbo Branch of Renji Hospital, Shanghai Jiao Tong University School of Medicine, Shanghai), Ningbo, Zhejiang, China; 3 The Affiliated Yanming Hospital of Ningbo University, Ningbo, Zhejiang, China

**Keywords:** fecal microbiota transplantation, gut-liver axis, high-fat and high-sugar diet, MAFLD, MASH

## Abstract

**Aim:**

To investigate whether fecal microbiota transplantation (FMT) could alleviate high-fat and high-sugar (HFCS) diet-induced metabolic dysfunction-associated fatty liver disease (MAFLD) in mice and explore potential mechanisms underlying gut microbiota modulation.

**Methods:**

A MAFLD mouse model was established by feeding mice a HFCS diet for 20 weeks, followed by an 8-week intervention with FMT or saline, continuing for a total of 28 weeks. Gut microbiota composition, serum biochemical markers, liver histopathology, and inflammatory cytokine expression were evaluated.

**Results:**

The HFCS diet induced significant changes in gut microbiota, including increased Firmicutes and decreased Bacteroidetes and Bifidobacterium. FMT partially restored microbiota composition to resemble that of control mice. Mice receiving FMT showed reduced body weight and a consistent trend toward improvement in serum alanine transaminase and total cholesterol levels, although these changes did not reach statistical significance. Liver histology showed amelioration of steatosis and inflammation, as evidenced by reduced MAFLD activity score and decreased intrahepatic expression of IL-1β and IL-17α mRNA. To further explore potential mechanisms, we analyzed a public liver transcriptomic dataset (GSE151220) involving FMT from dysbiotic donors. Differentially expressed genes were enriched in lipid metabolism and extracellular matrix-related pathways, processes known to be involved in MAFLD progression.

**Conclusion:**

These results suggest that FMT is associated with modulation of the gut–liver axis and partial alleviation of HFCS-induced MAFLD features in mice. FMT may serve as a potential adjunctive strategy for managing MAFLD.

## 1 Introduction

Metabolic dysfunction-associated fatty liver disease (MAFLD) encompasses a spectrum of liver disorders, including metabolic dysfunction-associated steatotic liver disease (MASLD), metabolic dysfunction-associated steatohepatitis (MASH), fibrosis, cirrhosis, and hepatocellular carcinoma (HCC) (NAFLD, 2022; Sharpton et al., 2019). The global prevalence of MASLD has been increasing rapidly, with current estimates showing that 38% of all adults and 7%–14% of children and adolescents are affected. By 2040, the prevalence rate for adults is projected to rise to more than 55% (Pouwels et al., 2022; Henry et al., 2022; Barritt, 2021; Younossi et al., 2025). Studies have shown that individuals in Asian and Western populations are susceptible to MAFLD even at normal body mass index (BMI), potentially due to excessive visceral fat accumulation (WHO Expert Consultation, 2004).

Although the first MASH-specific drug was recently approved by the FDA in 2024, microbiota-targeted therapies like fecal microbiota transplantation (FMT) remain relevant as adjunctive strategies (Powell et al., 2021). The gut–liver axis plays a central role in metabolic diseases. Accordingly, increasing attention has been directed toward the intestinal microbiota as both a pathogenic factor and a therapeutic target in MAFLD (Khoruts and Sadowsky, 2016). A positive balance between intestinal flora and the host is beneficial to the normal development of the biological metabolic system (Patterson et al., 2016). The human colon harbors trillions of microorganisms that not only aid digestion but also regulate immune responses and host metabolism (Aron-Wisnewsky et al., 2020). Disruption of this microbial ecosystem, commonly referred to as dysbiosis, has been implicated in the development and progression of MAFLD (Boursier et al., 2016; Y et al., 2019).

FMT is a microbiome-based therapeutic strategy involving the transfer of gut microbial communities from a healthy donor to a recipient in order to restore microbial balance (Matsuoka, 2021). FMT has shown promise in treating several microbiota-associated conditions, such as inflammatory bowel disease, recurrent Clostridioides difficile infection, autism, and metabolic syndrome (Gupta et al., 2021; Zhang et al., 2018). FMT has shown potential in improving liver function and metabolic health by modulating the gut microbiota (Ashiqueali et al., 2025). In the context of MAFLD, several studies have shown that FMT can restore the microbial ecosystem and reduce liver inflammation, thus providing a promising adjunct to conventional treatments (Lee et al., 2023; Xue et al., 2022). Compared to probiotics or synbiotics, FMT offers the advantage of delivering a more diverse and complex microbial population (Hu et al., 2020). While previous studies suggest that restoring gut microbiota homeostasis via FMT may benefit liver function and metabolic health in MAFLD (Chen and Vitetta, 2020), most studies have relied on antibiotic pretreatment to deplete the host microbiota before FMT, which introduces potential confounders such as microbial depletion-induced epithelial damage or altered immune responses (Yu et al., 2020). Furthermore, the effects of FMT without antibiotic pretreatment in the context of diet-induced fatty liver models have been less thoroughly investigated.

In this study, we employed a high-fat, high-sugar (HFCS) diet to induce MAFLD in C57BL/6 mice and examined whether FMT derived from healthy donors could reverse the hepatic and microbial alterations without the use of antibiotic pretreatment. Our objective was to evaluate the effects of FMT on liver inflammation, serum biochemistry, and gut microbial composition. Our results provide insight into the therapeutic potential of FMT in MAFLD.

## 2 Materials and methods

### 2.1 Animals experiments

All animal experiments were reviewed and approved by the Ethics Committee for Animal Research at the Ningbo University School of Medicine (Approval No. AEWC-NBU20210012) and performed in compliance with institutional regulations and the ARRIVE (Animal Research: Reporting of *In Vivo* Experiments) guidelines. Male C57BL/6 mice (*Mus musculus*, 5–6 weeks old, weighing 20 ± 2 g) were obtained from Zhejiang Weitong Lihua company and were maintained under specific pathogen-free (SPF) conditions at 21 °C–22 °C, with a 12-h light/dark cycle and unrestricted access to food and water. After a 1-week acclimatization period, the animals were randomly allocated into three experimental groups (n = 5 per group) and housed in separate cages to avoid cross-contamination of gut microbiota. Only male mice were included to minimize variability associated with hormonal cycling. All animals were of similar age at study initiation to reduce age-related confounding effects. Potential sex- and age-specific differences in MAFLD susceptibility were not addressed in this study and warrant further investigation.

The study included three groups: Control, HFCS, and HFCS + FMT. The HFCS diet was chosen due to its well-established ability to induce MAFLD in mice, mimicking the conditions seen in human MAFLD models. The Control group was fed a standard chow diet, while the HFCS and HFCS + FMT groups received a high-fat and high-sugar diet consisting of 60% kcal from fat and 42 g/L sugar water (55% fructose and 45% sucrose, w/w), which was purchased from Changzhou Shuyi Shuer Biotechnology Co., Ltd. A 28-week treatment period was selected to allow sufficient time for the development of metabolic disturbances and to evaluate the long-term effects of FMT intervention. After 20 weeks on the HFCS diet, mice in the Control and HFCS groups were gavaged with 200 μL of sterile saline, whereas the HFCS + FMT group was given freshly prepared fecal microbiota suspension (200 μL per gavage) three times per week for 8 weeks, totaling 24 administrations. For each FMT session, donor feces (200 mg) from healthy mice fed a standard chow diet were homogenized in 4 mL of sterile saline, vortexed for 5 min, passed through sterile gauze to remove large particles, and the clarified supernatant was collected for transplantation, following established protocols with slight modifications (Mi et al., 2023).

Body weight was measured weekly, and the overall health condition of the mice, including coat appearance and activity levels, was regularly assessed. At the conclusion of the 28-week experiment, fresh fecal samples were collected individually from each animal for subsequent microbiota profiling. Mice were then anesthetized using 40% carbon dioxide and sacrificed by cardiac puncture to obtain blood and tissue samples. Liver specimens were excised immediately after euthanasia. Approximately half of the largest hepatic lobe was immersed in 10% neutral-buffered formalin at a fixative-to-tissue volume ratio of 10:1 for histopathological examination, while the remaining portion was rapidly frozen in liquid nitrogen and stored at −80 °C for downstream molecular analyses.

### 2.2 Histological analysis

Formalin-fixed liver samples were paraffin-embedded, sectioned at a thickness of 4 μm, and subjected to hematoxylin and eosin (H&E, Beyotime, Beijing, China) staining using standard histological protocols. Liver morphology was examined under a light microscope, and the NAFLD activity score (NAS) was calculated based on the combined assessment of steatosis, lobular inflammation, and hepatocyte ballooning. While the NAS was originally developed for NAFLD, it remains a widely accepted histological scoring system and was applied here to assess disease activity in a MAFLD mouse model.

### 2.3 Biochemical analysis

Serum alanine aminotransferase (ALT) and total cholesterol (TC) concentrations were quantified using commercially available assay kits (Ningbo MedicalSystem Biotechnology Co., Ltd., China) in accordance with the manufacturer’s protocols.

### 2.4 Quantitative real-time PCR (qRT-PCR)

Total RNA was isolated from frozen liver tissues using TRIzol reagent (Invitrogen, United States). RNA integrity, purity, and concentration were evaluated with a NanoDrop spectrophotometer (Denovix, United States). First-strand complementary DNA (cDNA) was synthesized using the TransScript® All-in-One First-Strand cDNA Synthesis Kit (TransGen Biotech, Beijing, China). Quantitative real-time PCR (qRT-PCR) was conducted with SYBR Green-based detection. Amplification was performed using a reaction cycle at 94 °C 30 s, 94 °C 5 s, and then 60 °C 34 s, 40 cycles. Relative expression levels of *IL-1β* and *IL-17α* were normalized to *GAPDH* expression using the 2^−ΔΔCT^ method. Primer sequences for mouse *GAPDH*, *IL-17α*, and *IL-1β* are provided in Table 1.

**TABLE 1:** qRT-PCR primers.

Gene	Primers	Sequence
*GAPDH*	Sense	TGC​CAC​CTT​TTG​ACA​GTG​ATG
	Anti-sense	ATG​TGC​TGC​TGC​GAG​ATT​TG
*IL-17α*	Sense	GCC​CTC​AGA​CTA​CCT​CAA​CC
	Anti-sense	CAG​CTT​TCC​CTC​CGC​ATT​GA
*IL-1β*	Sense	TCC​CAG​CTT​AGG​TTC​ATC​AGG
	Anti-sense	TTT​GCC​GTG​AGT​GGA​GTC​AT

### 2.5 Gut microbiota analysis

The DNA of each group was extracted using the QIAamp Fast DNA Stool Mini Kit (Cat. No. 51604, Qiagen GmbH, Germany) according to the instructions. The V3-V4 region of the 16S rRNA gene was targeted for microbiota analysis. Primers 341F 5′-CCTACGGGRSGCAGCAG-3′ and 806R 5′-GGACTACVVGGGTATCTAATC-3′ with specific barcodes were used to amplify the V3–V4 region of the bacterial ribosomal RNA gene to obtain a ∼500 bp product. The PCR products were detected by 2% agarose gel electrophoresis, and the PCR products were recovered by cutting the gel using the AxyPrep DNA Gel Extraction Kit (Axygen Biosciences, CA, United States) according to the manufacturer’s instructions. Qubit 2.0 (Invitrogen, United States) was used for library quantification, and samples were mixed with corresponding ratios according to the data volume requirements. Paired-end sequencing was performed using the HiSeq platform PE250 strategy (Illumina, Inc., CA, United States). Long reads of hypervariable regions were assembled by the overlaps between raw reads. The 16S read sequence length is controlled between 220 and 500 bp. The average quality value of each read is larger than 20, and the number of Ns is not more than 3. Clean Reads with the same sequence were sorted according to their abundance. Singletons were filtered out. UPARSE (http://drive5.com/uparse/) was used for Operational Taxonomic Unit (OTU) clustering based on 97% similarity. USEARCH (version 7.0) was used to identify and remove chimeric sequences. A representative sequence for each OTU was obtained using the RDP database (http://rdp.cme.msu.edu/) with a confidence threshold of 0.8. The RDP Classifier was used to annotate species by each representative sequence. OTU profiling table and alpha/beta diversity analysis are implemented through Qiime’s Python script. Sample extraction, library construction, sequencing, and analysis services are completed by Shanghai Ruiyi Biotechnology Co., Ltd.

### 2.6 RNA-seq data analysis (GSE151220)

To investigate the transcriptomic consequences of microbiota-derived metabolic dysfunction and validate our experimental observations, we analyzed the public RNA-seq dataset GSE151220, which profiled liver gene expression in mice following FMT from donors with gut dysbiosis induced by dehydroepiandrosterone (DHEA) (Han et al., 2021). The analysis workflow is described as follows.

Raw gene expression data were imported from CSV files, and genes were pre-filtered by retaining those with both average expression and coefficient of variation (CV) above the 25th percentile. The selected genes were normalized using quantile normalization implemented in the preprocessCore R package. Differentially expressed genes (DEGs) were identified based on the thresholds: |log_2_ fold change| > 0.3 and adjusted p-value <0.05.

To visualize transcriptomic data, a volcano plot was generated. The top 10 DEGs were displayed using boxplots, and unsupervised hierarchical clustering was performed to create a heatmap of expression profiles. Functional enrichment analyses for Gene Ontology (GO) terms and KEGG pathways were carried out using the clusterProfiler package, and significantly enriched biological processes (p < 0.05) were visualized with dot plots. All analyses were conducted in R (v4.1.0 or higher) using the following packages: limma, preprocessCore, clusterProfiler, org.Hs.e.g.,.db, ggplot2, ggrepel, ggsignif, and tibble.

### 2.7 Statistical analysis

Data were expressed as mean ± standard error, with statistical analysis conducted using SPSS Statistics Version 26.0 software (IBM, China). Results were illustrated using GraphPad Prism9.0 software (GraphPad Software Inc., CA, United States). To compare differences between groups, a one-way ANOVA was conducted, followed by Tukey’s test for pairwise comparisons. A P-value of less than 0.05 indicates statistical significance.

## 3 Results

### 3.1 FMT reduced body weight and improved liver enzyme profiles in MAFLD mice

**FIGURE 1:**
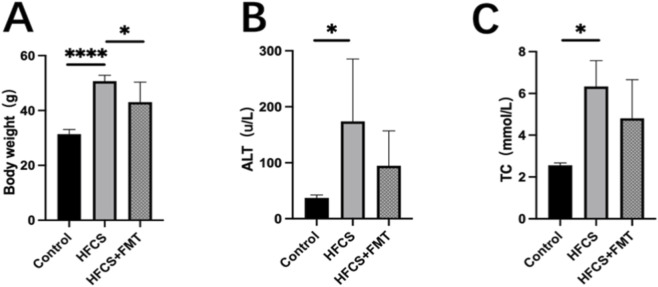
Effects of FMT on body weight, liver function, histopathology, and inflammatory markers in HFCS-fed mice. **(A)** Body weight at week 28. **(B,C)** Serum ALT and TC levels. Data are presented as mean ± SEM (n = 5 per group). *P < 0.05, **P < 0.01 and, ****P < 0.0001.

Mice in the HFCS group exhibited signs of obesity, lethargy, and coarse fur compared to the control group. Following 8 weeks of FMT intervention, the HFCS + FMT group showed improved general health, including reduced body weight and glossier fur. At the end of the 28-week experiment, body weight in the HFCS + FMT group was significantly lower than in the HFCS group (Figure 1A).

Serum ALT and TC levels were significantly elevated in the HFCS group compared to controls, indicating liver injury and dyslipidemia. Although FMT-treated mice exhibited a consistent trend toward reduced ALT and TC levels, these differences did not reach statistical significance (Figures 1B,C), likely reflecting limited statistical power due to sample size.

### 3.2 FMT modulated the composition of the gut microbiota

**FIGURE 2:**
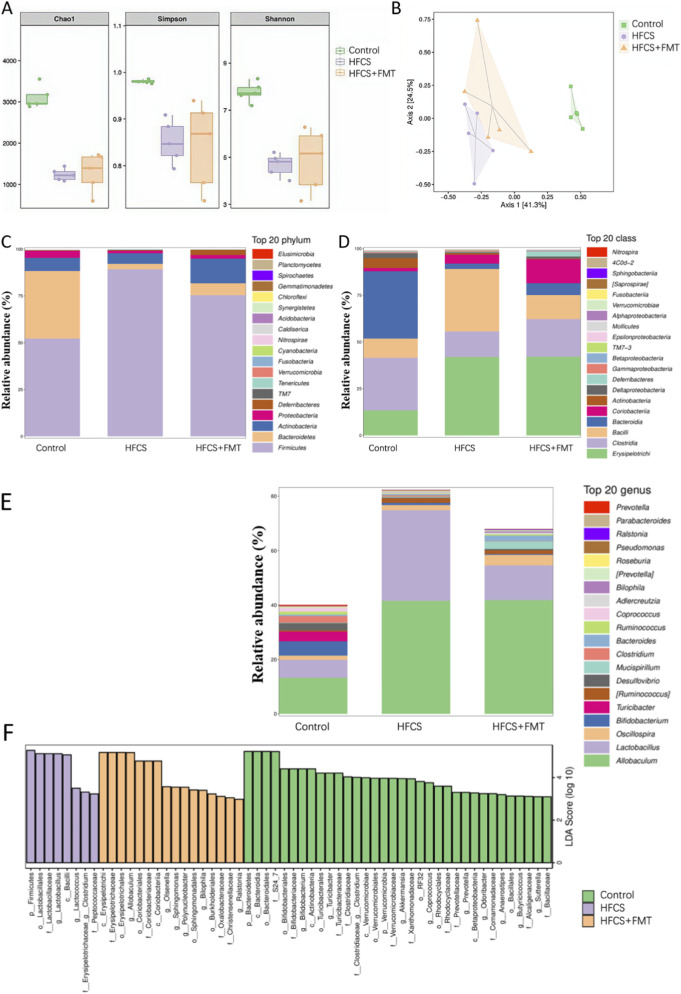
Improvement of intestinal flora by FMT (n = 5 mice per group). **(A)** Alpha-diversity analysis using Chao1, Shannon, and Simpson indices. **(B)** Principal coordinate analysis (PCoA). **(C)** Bacterial composition of the different communities at the phylum level. **(D)** Bacterial composition of the different communities at the class level. **(E)** Bacterial composition of the different communities at the genus level. **(F)** Histogram of the linear discriminant analysis (LDA) scores.

Fecal microbiota analysis was performed at the end of the 28-week study. To assess the diversity within the gut microbiota, alpha-diversity was evaluated using Chao1, Shannon, and Simpson indices. The analysis showed that the HFCS group had significantly lower Chao1, Shannon, and Simpson values compared to the Control group (Figure 2A). However, after FMT intervention, the HFCS + FMT group exhibited an increase in Chao1, Shannon, and Simpson values. These findings suggest that MAFLD mice have reduced gut microbiota diversity, and that FMT intervention can effectively restore gut microbiota diversity. Principal coordinate analysis (PCoA) based on 16S rRNA sequencing revealed distinct microbial community structures among the three groups. The HFCS group clustered separately from the control group, while the HFCS + FMT group shifted closer to the control cluster. These results suggest partial restoration of microbial composition after FMT (Figure 2B).

At the phylum level, the HFCS group showed an increased Firmicutes-to-Bacteroidetes ratio compared to controls, which was partially reversed by FMT (Figure 2C). At the class level, Bacilli were significantly enriched in the HFCS group (Figure 2D). At the genus level, Bifidobacterium was notably depleted in HFCS-fed mice but increased in the HFCS + FMT group (Figure 2E). Linear discriminant analysis effect size (LEfSe) identified key taxa that were differentially abundant between groups, further supporting the regulatory effect of FMT on gut microbiota composition (Figure 2F).

### 3.3 Functional pathway enrichment analysis of gut microbiota

**FIGURE 3:**
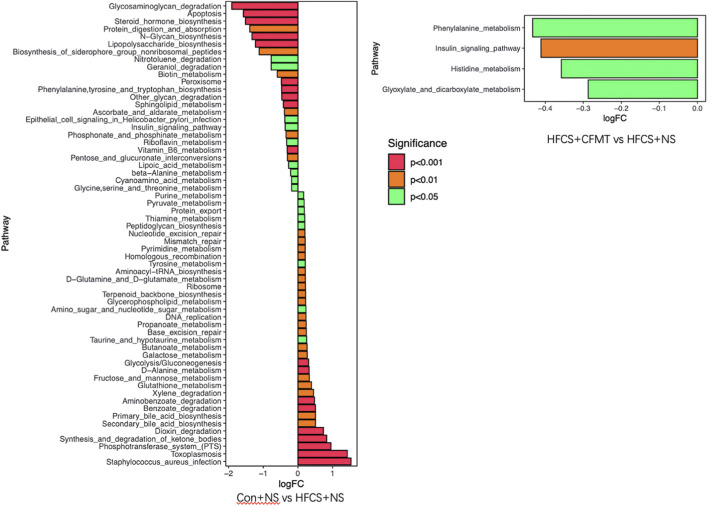
Differential metabolic pathways in HFCS vs. control, and HFCS + FMT vs. HFCS groups.

To explore potential functional differences in the gut microbiota following FMT intervention, microbial functional profiles were predicted using PICRUSt. Predicted pathways that differed both between the control and HFCS groups and between the HFCS and FMT groups were considered differentially enriched pathways. Among these, pathways related to insulin signaling were identified at the prediction level (Figure 3).

### 3.4 FMT alleviated hepatic steatosis and inflammation

**FIGURE 4:**
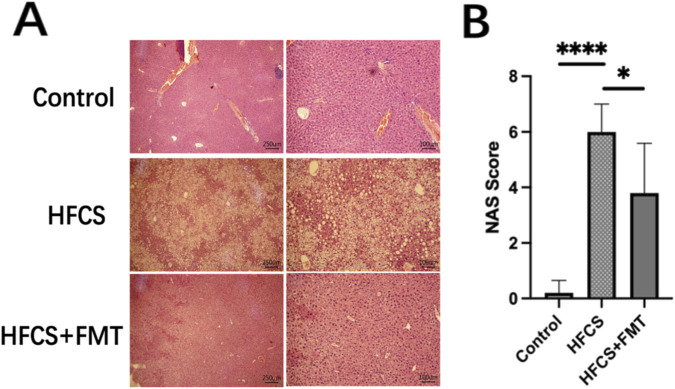
Effects of FMT on liver histopathology and inflammation in HFCS-fed mice. **(A)** Representative H&E-stained liver sections. **(B)** NAFLD activity score (NAS). Data are presented as mean ± SEM (n = 5 per group). *P < 0.05, **P < 0.01 and, ****P < 0.0001.

Histological examination of liver sections via H&E staining showed normal hepatic architecture in the control group, while the HFCS group displayed disrupted lobular structure, hepatocyte ballooning, and inflammatory cell infiltration. FMT treatment markedly improved liver histology, with reduced steatosis, fewer fat vacuoles, and more organized hepatic cords (Figure 4A). NAS was significantly higher in the HFCS group than in the control group, and was significantly reduced in the HFCS + FMT group (Figure 4B, P = 0.0348). These results demonstrate that FMT effectively attenuated HFCS-induced steatohepatitis.

### 3.5 FMT decreased hepatic inflammatory cytokine expression

**FIGURE 5:**
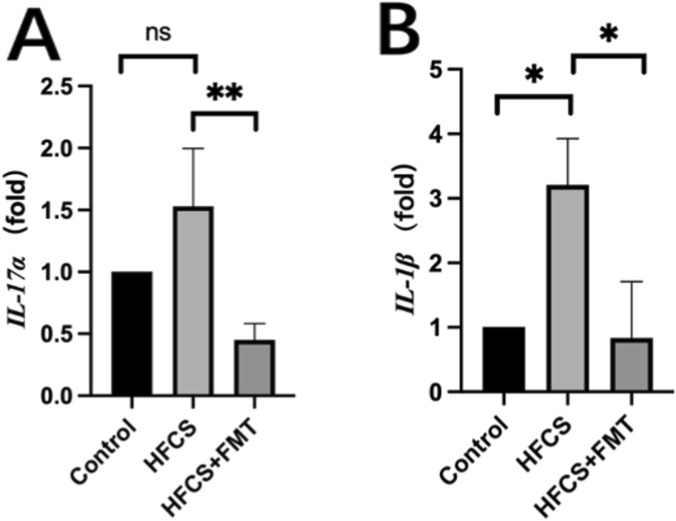
Effects of FMT on gene expression of liver inflammatory markers. **(A,B)** Relative hepatic mRNA expression of IL-1β and IL-17α. Data are presented as mean ± SEM (n = 5 per group). *P < 0.05, **P < 0.01 and, ****P < 0.0001.

To investigate the hepatic inflammatory response, we assessed mRNA expression of IL-1β and IL-17α in liver tissue. These two genes have been identified as critical proinflammatory cytokines in the regulation of both obesity and MAFLD pathogenesis (Giles et al., 2015). IL-1β mRNA levels were significantly upregulated in the HFCS group compared to the control group and were significantly reduced by FMT treatment (Figure 5A). Similarly, IL-17α mRNA levels were elevated in HFCS-fed mice but decreased following FMT (Figure 5B). These findings suggest that FMT is associated with reduced hepatic inflammation and proinflammatory cytokines.

### 3.6 Validation of the role of FMT in hepatic gene regulation

To further support our experimental findings and investigate the transcriptomic impact of microbiota manipulation on the liver, we analyzed the publicly available RNA-seq dataset GSE151220. It used mice that received FMT derived from donors with gut dysbiosis induced by DHEA, which differs substantially from HFCS-induced MAFLD. In contrast to our study, where FMT from healthy donors alleviated liver injury, this dataset represents the opposite scenario: FMT from dysbiotic donors associated with metabolic dysfunction. Therefore, these transcriptomic findings were used to provide contextual and supportive evidence rather than direct mechanistic validation of our experimental model.

**FIGURE 6:**
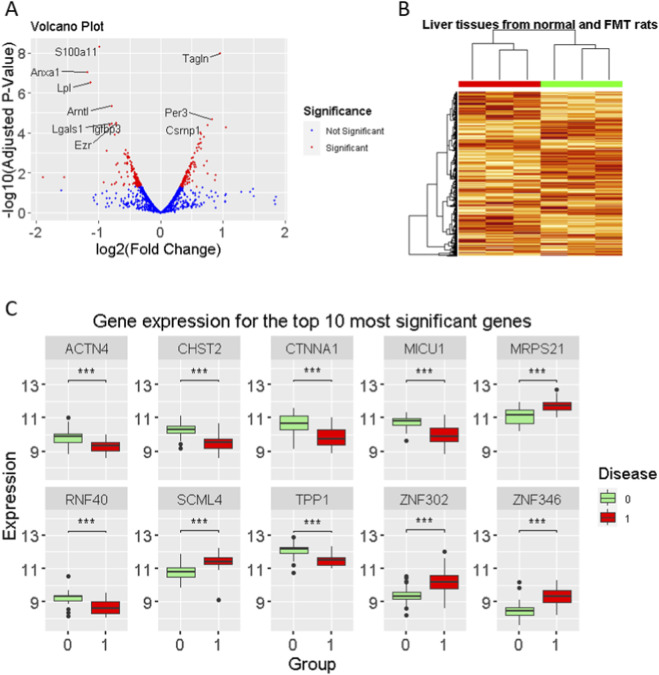
Transcriptomic alterations in mouse liver following dysbiotic FMT (GSE151220). **(A)** Volcano plot showing differentially expressed genes between dysbiotic FMT-treated mice and control mice. Red and blue dots indicate significantly upregulated (n = 118) and downregulated genes (n = 140), respectively (|log_2_FC| > 0.3, adjusted p < 0.05). **(B)** Heatmap with hierarchical clustering of differentially expressed genes demonstrates clear segregation between FMT and control groups. **(C)** Expression profiles of representative genes with the largest fold changes. Genes such as *MRPS21*, *SCML4*, *ZNF302*, and *ZNF346* were markedly upregulated, while *ACTN4*, *CHST2*, *CTNNA1*, *MICU1*, *RNF40*, and *TPP1* were significantly downregulated in the FMT group.

Differential expression analysis between FMT-treated mice and control mice revealed 118 upregulated and 140 downregulated genes (Figure 6A). Unsupervised clustering analysis showed that these gene expression patterns clearly distinguished between the two groups, highlighting a strong transcriptional response to dysbiotic FMT (Figure 6B). Notably, genes such as *MRPS21*, *SCML4*, *ZNF302*, and *ZNF346* were significantly upregulated, while *ACTN4*, *CHST2*, *CTNNA1*, *MICU1*, *RNF40*, and *TPP1* were markedly downregulated in the FMT group (Figure 6C).

**FIGURE 7:**
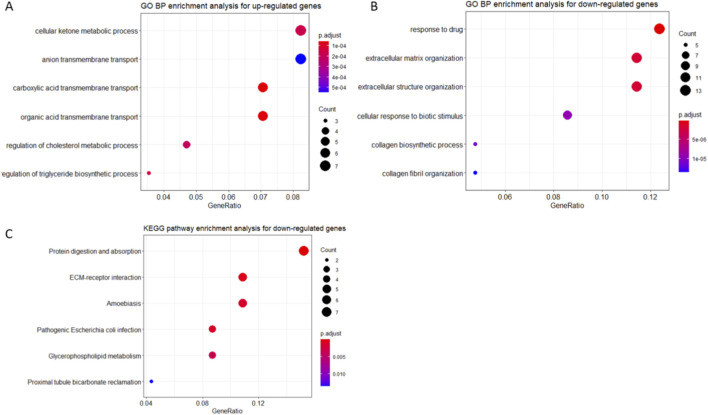
Functional enrichment of dysbiotic FMT-induced gene expression changes. **(A)** GO and KEGG enrichment analysis of upregulated genes. **(B)** GO terms enriched among downregulated genes. **(C)** KEGG pathway analysis of downregulated genes. Only terms with adjusted p-values <0.05 are shown. Bar lengths correspond to gene ratio (hits/total), and color denotes significance level.

GO and KEGG pathway enrichment analyses of the upregulated genes indicated significant enrichment in pathways related to cellular ketone metabolism, cholesterol metabolism, and triglyceride biosynthesis regulation (Figure 7A). In contrast, the downregulated genes were associated with drug response, extracellular matrix (ECM) organization, and cellular responses to biotic stimuli (Figures 7B,C)—biological processes that are well-recognized hallmarks of MAFLD pathogenesis.

## 4 Discussion

With global dietary shifts toward high-fat and high-sugar consumption, MAFLD has emerged as the most prevalent chronic liver disease. MAFLD is closely associated with obesity and insulin resistance (Gofton et al., 2023; Xian et al., 2020). In the present study, we established a mouse model of MAFLD using a HFCS diet, which closely mimics the pathophysiological features of human disease. Previous studies have demonstrated that gut dysbiosis plays a crucial role in the progression of MAFLD and MASH. Transplantation of gut microbiota from MASH patients into germ-free or antibiotic-treated mice exacerbates hepatic steatosis and inflammation, underscoring the pathogenic role of the microbiota (Chiu et al., 2017). However, most preclinical studies of FMT in MAFLD models rely on antibiotic pretreatment to eliminate endogenous flora, which may introduce confounding factors such as microbiota depletion-induced epithelial damage or immune alteration (Yu et al., 2020). Using broad-spectrum antibiotics before FMT might lead to side effects and pose ethical and antibiotic management challenges (Singh et al., 2022). In contrast, our study adopted a gentler and more translational approach without prior antibiotic treatment, thereby preserving the host’s native microbiota context and minimizing confounders.

Our results indicate that FMT was associated with partial reversal of HFCS-induced dysbiosis, characterized by a reduced Firmicutes-to-Bacteroidetes ratio and increased Bifidobacterium abundance. These microbial features have been consistently associated with metabolic status in obesity and MAFLD models (Indiani et al., 2018). The Firmicutes-to-Bacteroidetes ratio was identified as a possible biomarker of gut dysbiosis (Grigo r’eva IN, 2020). Previous studies have suggested that alterations in the Firmicutes-related microbial balance may serve as indicators of obesity-related metabolic dysregulation (Zou et al., 2020; Bervoets et al., 2013). Fecal Bacilli, significantly enriched in HFCS, was reported to be positively correlated with serum fatty acids in cafeteria diet fed rat. However, role Bacilli in MAFLD is not fully explored (de la Garza et al., 2022). Bifidobacterium, a beneficial genus depleted in HFCS, was restored in the FMT-treated mice. Previous studies have shown that Bifidobacterium can reduce intestinal permeability and inflammation, suggesting its pivotal role in maintaining metabolic health (Turroni et al., 2011; Azad et al., 2018).

To support the biological relevance of our findings, we analyzed a public RNA-seq dataset (GSE151220) in which mice received FMT from metabolically unhealthy donors. In contrast to our model using healthy donors, dysbiotic FMT in this dataset was associated with hepatic transcriptomic alterations related to lipid metabolism and extracellular matrix organization—processes implicated in MAFLD pathogenesis. The FMT from the lean donor to obese patients led to sustained changes in the intestinal microbiome and bile acid profiles that were similar to those of the lean donor (Allegretti et al., 2020). These observations highlight the influence of donor microbiota composition on hepatic gene expression and provide contextual, indirect support for the potential benefits of healthy-donor FMT observed in our study.

Histological analysis showed that FMT intervention reduced steatosis and lobular inflammation, reflected by decreased NAS scores. This was accompanied by downregulation of hepatic proinflammatory cytokines *IL-1β* and *IL-17α*, of which *IL-17α* is a potential driver in MAFLD progression (Giles et al., 2015). These results suggest the associations among FMT, alleviating liver injury, and immunomodulation of the gut-liver axis. However, these findings are based solely on transcriptional measurements. Without protein-level validation or immune cell profiling, conclusions regarding immunomodulatory mechanisms should be interpreted cautiously.

In addition, our study shows that long-term FMT without antibiotic pretreatment can improve liver pathology and reshape gut microbiota in a diet-induced MAFLD model. It was reported that MAFLD disease progression greatly varies across different strains (Hui et al., 2018). C57BL/6 mice were selected due to their widespread use and extensive characterization in diet-induced MAFLD studies. Nevertheless, other strains may exhibit distinct metabolic responses, and strain-dependent effects cannot be excluded. Furthermore, species-specific differences in lipid metabolism, immune regulation, and gut microbiota composition limit direct translation of murine findings to humans. Thus, the present results should be viewed as hypothesis-generating rather than directly predictive of clinical efficacy.

Only male mice were included in this study to minimize variability associated with the estrous cycle and to ensure consistency in metabolic phenotyping. Importantly, accumulating evidence indicates that susceptibility to diet-induced metabolic liver disease differs substantially between sexes. Previous studies have shown that, under high-fat or “fast-food” dietary conditions, male rodents tend to develop more severe hepatic lipid accumulation, hepatomegaly, and early fibrotic features, whereas females preferentially accumulate lipids in adipose depots with relatively attenuated liver involvement (Meyer et al., 2025). In addition, sex-specific inflammatory responses have been reported, with males exhibiting more pronounced fibrotic progression, while females display distinct inflammatory profiles, including higher expression of cytokines such as IL-1β and IFN-γ and enhanced inflammasome activity (Kucsera et al., 2021). Moreover, underlying molecular mechanisms also differ by sex; for example, activation of antioxidant pathways such as NRF2 during early steatosis has been observed in females but not in males in diet-induced models (Di Veroli et al., 2024). In light of these established sex-dependent differences, the exclusive use of male mice in the present study was intended to reduce biological heterogeneity and to focus on a phenotype with higher susceptibility to hepatic injury in MAFLD models. Nevertheless, this design choice limits the generalizability of our findings, and future studies incorporating both sexes will be essential to fully characterize sex-specific responses to FMT-based interventions.

Furthermore, our study did not include lipid-specific (e.g., Oil Red O) or fibrosis-specific (e.g., Masson’s trichrome) staining to evaluate hepatic fat accumulation and fibrogenesis. The absence of a detailed assessment of steatosis grade and fibrosis represents a limitation of this work. Therefore, histological conclusions should be considered preliminary. The relatively small sample size limited statistical power, particularly for serum biochemical parameters such as ALT and TC. Although FMT showed a trend toward improved ALT and TC levels, these changes were not statistically significant. This may be due to the short duration of FMT treatment (28 days), which might not be sufficient to produce significant changes in serum biochemistry. Accordingly, these findings should be interpreted as indicative trends rather than definitive evidence. We did not directly quantify engraftment efficiency at the strain level. Successful microbiota transfer was inferred from global compositional shifts rather than direct donor strain tracking, which represents another limitation of the current study. Future studies incorporating these parameters and larger sample sizes will be essential to validate the therapeutic efficacy of FMT.

In conclusion, this study demonstrates that long-term FMT without antibiotic pretreatment is associated with partial improvement of gut microbiota composition and liver pathology in a HFCS-induced MAFLD mouse model. While histological and inflammatory markers were favorably altered, biochemical and mechanistic findings remain exploratory due to limited sample size and methodological constraints. These results support further investigation of microbiota-targeted interventions as adjunctive strategies for MAFLD.

## Data Availability

The 16S rRNA sequencing data used in this study are available in the NCBI Sequence Read Archive (SRA) under the accession number PRJNA1344756. The data can be accessed at https://www.ncbi.nlm.nih.gov/bioproject/PRJNA1344756.
